# Fast Expansion of the Asian-Pacific Genotype of the Chikungunya Virus in Indonesia

**DOI:** 10.3389/fcimb.2021.631508

**Published:** 2021-04-21

**Authors:** Yusnita Mirna Anggraeni, Triwibowo Ambar Garjito, Mega Tyas Prihatin, Sri Wahyuni Handayani, Kusumaningtyas Sekar Negari, Ary Oktsari Yanti, Muhammad Choirul Hidajat, Dhian Prastowo, Tri Baskoro Tunggul Satoto, Sylvie Manguin, Laurent Gavotte, Roger Frutos

**Affiliations:** ^1^ Institute for Vector and Reservoir Control Research and Development, National Institute of Health Research and Development, the Ministry of Health of Indonesia, Salatiga, Indonesia; ^2^ HSM, University of Montpellier, CNRS, IRD, Montpellier, France; ^3^ Department of Parasitology, Faculty of Medicine, Public Health and Nursing, Gadjah Mada University, Yogyakarta, Indonesia; ^4^ Espace-DEV, University of Montpellier, Montpellier, France; ^5^ Cirad, UMR 17, Intertryp, Montpellier, France

**Keywords:** chikungunya, *Aedes aegypti*, *Aedes albopictus*, genotyping, Indonesia

## Abstract

Chikungunya is repeatedly affecting Indonesia through successive outbreaks. The Asian genotype has been present in Asia since the late 1950s while the ECSA-IOL (East/Central/South Africa - Indian Ocean Lineage) genotype invaded Asia in 2005. In order to determine the extension of the circulation of the chikungunya virus (CHIKV) in Indonesia, mosquitoes were collected in 28 different sites from 12 Indonesian provinces in 2016-2017. The E1 subunit of the CHIKV envelope gene was sequenced while mosquitoes were genotyped using the mitochondrial *cox*1 (cytochrome C oxidase subunit 1) gene to determine whether a specific population was involved in the vectoring of CHIKV. A total of 37 CHIKV samples were found in 28 *Aedes aegypti*, 8 *Aedes albopictus* and 1 *Aedes butleri* out of 15,362 samples collected and tested. These viruses, like all Indonesian CHIKV since 2000, belonged to a genotype we propose to call the Asian-Pacific genotype. It also comprises the Yap isolates and viruses having emerged in Polynesia, the Caribbean and South America. They differ from the CHIKV of the Asian genotype found earlier in Indonesia indicating a replacement. These results raise the question of the mechanisms behind this fast and massive replacement.

## Introduction

The chikungunya virus (CHIKV) is an arbovirus, member of the genus *Alphavirus* in the family *Togaviridae*. CHIKV is a positive-strand RNA virus, 60-70 nm in diameter, spherical, and enveloped. The genome length is ca.12 kb, capped in 5’, with a polyA tail in the 3’ end. The genome has two open reading frames encoding two polyproteins: a non-structural polyprotein and a structural polyprotein. The polyproteins can be cleaved by proteases into four non-structural proteins: nsP1, nsP2, nsP3, nsP4; and five structural proteins (C, E3, E2, 6K, E1) (Higashi et al., 1967; Simizu et al., 1984; Powers et al., 2001; Khan et al., 2002). CHIKV was isolated for the first time in the Mokande plateau between Tanzania and Mozambique, in the Eastern part of the current Democratic Republic of Congo in 1958 (Osterrieth and Blanes-Ridaura, 1960; Osterrieth et al., 1961). Since then, chikungunya outbreaks were documented in South Africa, and Zimbabwe in 1956-1977; the Democratic Republic of Congo, Zambia, Senegal, Uganda, Nigeria, Angola and Republic of Central Africa in 1958-2000; and Equatorial Guinea in 2002 (Jupp and McIntosh, 1988; Powers and Logue, 2007; Desdouits et al., 2015; Zeller et al., 2016). CHIKV was introduced in Asia at two different times. The first expansion in Asia corresponded to the initial divergence of the Asian genotype from the ECSA (East/Central/South African) genotype, itself having diverged from the West African genotype (Hammon et al., 1960; Powers et al., 2000; Caglioti et al., 2013; Lo Presti et al., 2014). Chikungunya was for the first time reported in Asia, specifically in Thailand, in 1958 and early 1960 (Hammon et al., 1960; Weaver and Lecuit, 2015). The second Asian expansion corresponded to the emergence of the ECSA A226V mutant, also known as the Indian Ocean Lineage or IOL, which evolved from the ECSA genotype in Kenya in 2004 (Tsetsarkin et al., 2011; Coffey et al., 2014).

This re-emerging CHIKV, which displayed a higher affinity for *Aedes albopictus* and more precisely changes in the extrinsic incubation period (Tsetsarkin et al., 2007; Tsetsarkin and Weaver, 2011), became even more transmissible and virulent after additional mutations in Kerala, India (Tsetsarkin and Weaver, 2011; Agarwal et al., 2016). It spread eastward to cover continental Southeast Asia (Sam et al., 2009; Rianthavorn et al., 2010; Tsetsarkin et al., 2011; Duong et al., 2012). Currently, the ECSA-IOL genotype, circulates in many Asian countries (Sam et al., 2012; Chen et al., 2016; Sam, 2018).

In order to map the presence of vector-borne diseases and potential vectors in Indonesia, the Ministry of Health launched a large program of screening throughout the country. Part of the program was to identify which chikungunya virus was circulating in Indonesia, ECSA-IOL or Asian, or both, and where, and to genotype the vectors most likely involved in this circulation. We report in this article the results of this screening and the characterization of a genotype present all over Indonesia, which we propose to name the Asian-Pacific genotype.

## Materials and Methods

### Sampling Sites

In 2016-2017, larvae suspected to be *Ae. aegypti* and *Ae. albopictus* mosquitoes were collected from the field as previously described (IVRCRD, 2016) in 28 locations in 12 different Indonesian provinces: Riau, Riau Islands, Banten, Yogyakarta, Central Kalimantan, East Kalimantan, South Sulawesi, Southeast Sulawesi, Central Sulawesi, Maluku, North Maluku, Southeast Maluku, and West Papua (**Supplementary Tables 1**, **2**, **Supplementary Figure 1**). In this work only the samples used for CHIKV detection were considered. Three additional virus samples from human patients were obtained from the National Chikungunya Survey Program after a chikungunya outbreak in Magelang, province of Jateng (Central Java) in 2014 (**Table 1**). This sampling program was part of a larger initiative from the Ministry of Health to map viruses and vectors in Indonesia. The same standardized sampling methods were thus used for collecting mosquitoes for each objective, i.e. each individual virus (IVRCRD, 2016).

**Table 1:** Description of the samples used for sequencing.

Virus		CHIKV-positive *Aedes*		Location	
**Virus**	**ChikV code**	**Species**	**Sample**	**District**	**Province**
ChikV	a023	***Aedes albopictus***	**14_Aal**	Muna	South east Sulawesi
ChikV	a027	***Aedes aegypti***	**15_Aae**	Muna	South east Sulawesi
ChikV	a30	***Aedes aegypti***	**16_Aae**	Muna	South east Sulawesi
ChikV	a044	***Aedes aegypti***	**17_Aae**	Muna	South east Sulawesi
ChikV	10	***Aedes aegypti***	**9-18_Aae**	Fak-Fak	West Papua
ChikV	12	***Aedes butleri***	**012_Abt**	Bengkalis	Riau
ChikV	85	***Aedes aegypti***	**8_Aae**	South east Maluku	Maluku
ChikV	a070	***Aedes aegypti***	**6_Aae**	South east Maluku	Maluku
ChikV	a150	***Aedes albopictus***	**5_Aal**	South east Maluku	Maluku
ChikV	b084	***Aedes aegypti***	**3_Aae**	South east Maluku	Maluku
ChikV	a20	***Aedes aegypti***	**37_Aae**	South east Maluku	Maluku
ChikV	a024	***Aedes aegypti***	**15-18_Aae**	South Halmahera	North Maluku
ChikV	a050	***Aedes aegypti***	**13-11_Aae**	South Halmahera	North Maluku
ChikV	a072	***Aedes aegypti***	**19_Aae**	South Halmahera	North Maluku
ChikV	a085	***Aedes albopictus***	**22-30_Aal**	South Halmahera	North Maluku
ChikV	a71	***Aedes aegypti***	**2_Aae**	Lebak	Banten
ChikV	a078	***Aedes aegypti***	**ktg_H05_Aae**	Pulang Pisau	Central Kalimantan
NA	NA	***Aedes aegypti***	**10_Aae**	Bantul	Yogyakarta
		** CHIKV-negative *Aedes***		**Location**	
		**Species**	**Sample**	**District**	**Province**
		***Ae.aegypti***	**pl1_Aae**	Palu	Central Sulawesi
		***Ae.aegypti***	**pl26_Aae**	Palu	Central Sulawesi
		***Ae.albopictus***	**sls11_Aal**	Maros	South Sulawesi
		***Ae.albopictus***	**sls16_Aal**	Maros	South Sulawesi
		***Ae.aegypti***	**19_18_1_Aae**	Fak-Fak	West Papua
		***Ae.aegypti***	**tb131_Aae**	Batam	Riau islands
		***Ae.aegypti***	**mlk38_Aae**	Ambon	Maluku
		***Ae.aegypti***	**mlk40_Aae**	Ambon	Maluku
		***Ae.aegypti***	**mlk73_Aae**	Ambon	Maluku
		***Ae.aegypti***	**mlk654_Aae**	Ambon	Maluku
		***Ae.aegypti***	**mlk48_Aae**	Ambon	Maluku
		***Ae. aegypti***	**15-18_1_Aae**	South Halmahera	North Maluku
		***Ae. aegypti***	**13_11_1_Aae**	South Halmahera	North Maluku
		***Ae. aegypti***	**19_1_Aae**	South Halmahera	North Maluku
		***Ae. albopictus***	**ktg28_Aal**	Pulang Pisau	Central Kalimantan
		***Ae. aegypti***	**11_Aae**	Serang	Banten
		***Ae.aegypti***	**blp3_Aae**	Balikpapan	East Kalimantan
		***Ae.aegypti***	**jgj5_Aae**	Bantul	Yogyakarta

### Mosquito Collection

Collected larvae of *Ae. aegypti* and *Ae. albopictus* were reared individually until emergence of adults and then identified using standard taxonomic keys for *Stegomyia* (Rueda, 2004). Adults of *Aedes butleri* were collected by morning resting. Samples were segregated according to locality and date. Adult mosquitoes were stored individually in 1.5 ml microtubes with RNALater and stored at -80°C. Aliquots (head and thorax) were pooled for up to 25 individuals in 1.5-ml microtubes containing 250 µl of RNALater (Ambion-Thermo Fisher Scientific, Waltham, USA) to facilitate virus screening and minimize the number of RT-PCR reactions and stored at -80°C until further analysis. The pooling was conducted as follows:

1. Mosquitoes were stored individually in RNAlater.
2. An aliquot sample of each mosquito (head and thorax) was taken and the rest of the mosquito was kept individually.
3. Aliquot samples were pooled by up to 25 based on location and species. Specimen from different species or different locations were not mixed in the same pool.
4. An RT-PCR detection was ran on the pools.
5. When a pool was positive for CHIKV, a second aliquot sample of each of the mosquitoes making this pool was taken but this time each sample was analyzed individually by RT-PCR for the presence of CHIKV.

### Chikungunya Virus Detection

Chikungunya virus detection was carried out by one-step RT-PCR, selective for the E1 gene as previously described (Gopal, 2016). Excised head and thorax of each mosquito were homogenized in a sterile homogenizer and RNAs were extracted by the silica-based methods (RNA-easy minikit, Qiagen, Hilden, Germany). A single-step RT-PCR was performed using consensus primers (CHIK1: 5’-ACC GGC GTC TAC CCA TTC ATG T-3’; CHIK2: 5’GGG CGG GTA GTC CAT GTT GTA GA-3’). Master mix was prepared using Superscript III on step RT-PCR with platinum *Taq* DNA polymerase (Invitrogen, Life Technologies, Carlsbad, USA). The products were electrophoresed in alternate lanes in 2% agarose gels. Amplicons, 330-bp in size, were purified using Applied Biosystems ExoSAP-IT™ (Thermo Fisher Scientific, Vilnius, Lithuania). The detection was first conducted on each pool and, for the positive pools only, a second detection was done on each of the individual specimen present in these pools to identify those bearing the virus.

### Mosquito Genotyping

In order to map the different genotypes of potential mosquito vectors, the sequencing of the *cox1* (cytochrome c oxidase subunit) mitochondrial gene was performed. The amplification of the *cox*1 gene was conducted using the primers CI-N-2087 (5’-AAT TTC GGT CAG TTA ATA ATA TAG-3’) and TY-J-1460 (5’-TAC AAT TTA TCG CCT AAA CTT CAG CC-3’) as previously described (Rueanghiran et al., 2011). PCR reactions were carried out using the GoTaq^®^ Green Master Mix (Promega, Madison, WI, USA). The conditions for PCR amplification of the *cox*1 gene were as follows: 1 cycle at 94°C for 1 min for initial denaturation, followed by five cycles of 94°C for 30 s, 45°C for 40 s and 72°C for 1 min. This was then followed by 35 cycles of 94°C for 30 s, 44°C for 40 s and 72°C for 1 min, and by a final extension step at 72°C for 10 min (Rueanghiran et al., 2011). The products were electrophoresed in alternate lanes in 2% agarose gels. Amplicons, 650-bp in size, were purified using Applied Biosystems ExoSAP-IT™ (Thermo Fisher Scientific, Vilnius, Lithuania).

### DNA Sequencing

Cycle sequencing was performed using the primers listed above and an Applied Biosystems BigDye™ Terminator v.3.1 Cycle Sequencing Kit (Life Technologies Cooperation, Austin, TX, USA). To remove unincorporated BigDye^®^ terminators and salts, cycle sequencing products were purified using a BigDye^®^ Xterminator Purification Kit (Life technologies, Bedford, MA, USA). Sequence data were obtained using a DNA sequencer (Applied Biosystems^®^ 3500 Genetic Analyzer) and analyzed using the Sequencing Analysis 6 program (Applied Biosystems). All sequences have been deposited in Genbank under the accession numbers MW265951 to MW265970, MW270068 to MW270085 and MW270131 to MW270147.

### Phylogenetic Analyses

Sequences were aligned using MUSCLE in the SeaView package (Gouy et al., 2010). Multiple alignments and phylogenetic analyses were performed using the SeaView package (Gouy et al., 2010). The *Aedes cox*1 gene tree was built using the maximum likelihood method under the GTR+G model with 1,000 bootstrap repeats. The CHIKV E1 gene tree was built using the maximum likelihood method under the GTR model with 1,000 bootstrap repeats. Proteins trees were built using the maximum likelihood method under the LG model with 500 bootstrap repeats.

### Polymorphism Analyses

Polymorphism was analyzed using DNASp 6.12.03 (Rozas et al., 2017). The parameters considered were N: Number of samples, h: Number of haplotypes, S: Number of polymorphic sites, η: (Eta) Number of mutations, π: (Pi) Polymorphism at the nucleotide level, θ: (Theta) Watterson estimator of polymorphism (from Eta), ηs: Number of singletons, ηp: Number of parsimony significant sites, Ka/Ks ratio, Tajima’s D statistics, Fu and Li D* statistics and Fu and Li F* statistics. The haplotype relationship was computed and constructed as a distance map based on median-joining network using PopART version 1.7 (http://popart.otago.ac.nz/) (Bandelt et al., 1999; Clement et al., 2002; French et al., 2014).

## Results

### Mosquito Sampling and Chikungunya Virus Detection

Mosquito collections were conducted in 28 different locations in 12 provinces and a total of 15,362 mosquitoes, 11,776 *Ae. aegypti*, 3,421 *Ae. albopictus* and 165 *Ae. butleri* were collected (**Supplementary Table 1**). Other *Aedes* species were found, i.e. *Aedes lineatopennis, Aedes parasimilis, Aedes vexans, Aedes* sp. (*Verallina* sp.), *Aedes amesti, Aedes andamanensis, Aedes aurantius, Aedes poicilius, Aedes flavipennis, Aedes quadrifolium,* and *Aedes longirostris*. However, with the exception of one *Ae. butleri* individual from Bengkalis in the province of Riau, all were negative for CHIKV (**Supplementary Table 1**). *Aedes aegypti* was more frequent than *Ae. albopictus* in 17 locations, whereas *Ae. albopictus* was more frequent in 6 sampling sites (**Supplementary Table 1**). Only one place, Kulon Progo in Yogyakarta province, did not yield any *Ae. aegypti* sample and only one place, Bombana in Southeast Sulawesi, did not yield any *Ae. albopictus* sample (**Supplementary Table 1**). Mosquitoes were pooled by species and location for up to 25 individuals. A total of 1,190 pools tested for CHIKV, 800 pools (11,776 mosquitoes) for *Ae. aegypti*, 354 pools (3,421 mosquitoes) for *Ae. albopictus* and 36 pools (165 mosquitoes) of *Ae. butleri*. CHIKV was found in 25 *Ae. aegypti*, 8 *Ae. albopictus* and 1 *Ae. butleri* pools. Out of 33 pools of *Ae. aegypti* from Pulang Pisau (Central Kalimentan) only one was CHIKV-positive, but 4 out of the 25 mosquitoes making this pool were CHIKV-positive. All other CHIKV-positive pools contained only one CHIKV-infected mosquito (**Supplementary Table 1**). A total of 37 mosquitoes from these positive pools were found infected with CHIKV, including 28 *Ae. aegypti*, 8 *Ae. albopictus*, and 1 *Ae. butleri* (**Supplementary Table 1**). The infection rate (IR) was 0.24%, 0.23% and 0.6% for *Ae. aegypti*, *Ae. albopictus*, and *Ae. butleri*, respectively. However, owing to the limited number of specimens obtained for *Ae. butleri* and the difference in collection method, the IR for this species must be considered with caution.

### cox1 Phylogeny of *Aedes aegypti* and *Aedes albopictus* Mosquitoes

The *cox*1 genes of 29 *Ae. aegypti* samples (14 CHIKV-positive and 15 CHIKV-negative) and of 6 *Ae. albopictus* mosquitoes (3 CHIKV-positive and 3 CHIKV-negative) were sequenced (**Table 1**). No correlation was found for *Ae. aegypti* between the cox1 haplotype and the presence of CHIKV. The *cox*1 phylogenetic tree of *Ae. aegypti* mosquitoes showed a monophyletic topology with limited structuration (**Supplementary Figure 2**). The CHIKV-positive sample ktg_H05_Aae from Pulang Pisau in Central Kalimantan branched separately with a bootstrap of 70. The samples mlk48_Aae and mlk654 from Maluku clustered separately with a bootstrap of 57. Finally, a group comprising the CHIKV-negative samples mlk73_Aae, 11_Aae and the CHIKV-positive mosquitoes 3_Aae, 6_Aae, 10_Aae, 15_Aae, 37_Aae, 9-18_Aae and 13-11_Aae separated with a bootstrap value of 63 (**Supplementary Figure 2**). With the exception of 11_Aae from Banten (West Java), all other samples from this last group were from Eastern Indonesia, i.e. Southeast Sulawesi, Maluku and West Papua (**Table 1**). *Aedes albopictus* samples fell into two different clusters depending on whether they were CHIKV-positive or -negative with a rather high bootstrap value, i.e. 85 (**Supplementary Figure 2**). However, the number of samples is too low to draw any trend and reach any significant conclusion.

### Envelope Gene (E1) Phylogeny of the Isolated Chikungunya Viruses

The 17 CHIKV samples from mosquitoes collected in 2016-2017 for this work were included in a phylogenetic analysis with the 3 human CHIKV samples from Magelang/Jateng collected in 2014, with all CHIKV sequences from Indonesia present in Genbank, and sequences from the Asian, ECSA and West African genotypes (**Supplementary Figure 3**, **Supplementary Table 2**). All samples from this work clustered into two related populations derived from the Asian genotype (**Supplementary Figure 3**). A first population, cluster 1, comprised the samples 9C, a023, a027, a044, a070, a072, a078, a085, b084, a30 and a71. The second population, cluster 2, comprised the samples 10, 12, 85, a20, a150, a024 and a050. These clusters were characterized by weak bootstraps indicating that they were not genetically distinct from each other (**Supplementary Figure 3**). They corresponded to different locations and mosquito species, including *Ae. butleri*, suggesting that they were not linked to specific geographical clusters or vector populations. The three viruses isolated from human patients in 2014 did not correspond to the exact same populations as the mosquito samples but were very close. The samples B14 and 8F from clinical cases in Magelang, Province of Jateng, Central Java, in 2014 were identical to human isolates from totally different provinces, such as Banten, Bali and Jambi collected 2011, 2014 and 2015, respectively (**Supplementary Figure 3**, **Table 1**, **Supplementary Table 2**). The sample C7 from Magelang, Province of Jateng, Central Java, obtained in 2014 was related, but still different, to human samples from the Eastern tip of Java (East Java) in 2011 and to the cluster 2 of mosquito samples from this work (**Supplementary Figure 3**, **Supplementary Table 2**). When compared to the Asian genotype reference sequences, the E1 gene sequences from this work, and almost all Indonesian CHIKV sequences from Genbank, grouped within a single population comprising also CHIKVs from Yap Island, French Polynesia, Caribbean islands and South America (**Supplementary Figure 3**, **Supplementary Table 2**). This population was genetically distinct from known Asian genotype reference sequences (**Supplementary Figure 3**, **Supplementary Table 2**). Within the ECSA references, two 2011 Indonesian samples from West Kalimantan (KJ729851, KJ729852), one Indonesian sample from unknown exact location (KC862329) and five samples from Taiwan having been imported from Indonesia (KU561427, KU561428, KU561430, KU561431, KU561432) grouped with a CHIKV isolate of 2009 from Malaysia (HQ148971) and a 2011 isolate from Cambodia (JQ861254). They were all identical to the 2009 Malaysian sample suggesting the occurrence of imported cases (**Supplementary Figure 3**, **Supplementary Table 2**). The West African (HM045785) references (HM045785, MK028837, JQ943720) branched as expected in an ancestral position (**Supplementary Figure 3**, **Supplementary Table 2**). The sequences from this work as well as several reference sequences from Indonesia were truncated gene version and in particular did not cover the amino acid 226, which distinguishes ECSA from ECSA-IOL. Therefore, a full-length gene phylogenetic analysis was conducted with Indonesian samples and reference sequences from the ECSA, ECSA-IOL, Asian and West African (**Supplementary Figure 5**) present in Genbank. This full-length gene analysis confirmed what was seen with the truncated gene. The ancestral genotype is the West African genotype from which the ECSA genotype emerged (bootstrap = 98). The Asian genotype emerged from the ECSA genotype (bootstrap = 55) and gave rise to the Asian-Pacific genotype (bootstrap = 96). The ECSA-IOL genotype emerged independently from the ECSA genotype (bootstrap = 90). The haplotype mapping provided also a clear view of the evolution the different genotypes and the divergence of the Asian-Pacific genotype from the Asian one (**Supplementary Figure 5**). The analysis of polymorphism confirmed the existence of the Asian-Pacific as a population displaying a specific dynamic (**Table 2**). The Asian-Pacific genotype displayed a Pi value, which represents the polymorphism at the nucleotide level, clearly lower than that of the Asian genotype, even though the number of samples was far higher (98 vs. 7) (**Table 2**). The Tajima’s D, Fu and Li’s D* and Fu and Li’s F* statistics of the Asian-Pacific genotype were -1.50497, -3.25593, -3.04089, respectively, indicating a selective sweep and a population in expansion. In particular, the Fu and Li’s F* statistics displayed a value of -3.04089 significant at 0.02 which is indicative of a population in recent active expansion. The ECSA-IOL genotype displayed a similar trend, indicating also a population in expansion (**Table 2**). The full-length protein tree yielded the same topology as the gene phylogeny (**Supplementary Figure 6**). Four discriminating mutations, i.e. mutations linked to genotypes, were present (**Supplementary Figure 7**). The position 98 was characterized by an alanine (A) for the African genotypes, i.e. West African, ECSA and ECSA-IOL, and a threonine (T) for the Asian genotypes, i.e. Asian and Asian-Pacific. The position 145 was discriminative for the Asian-Pacific genotype, which was characterized by an alanine (A) whereas the Asian genotype displayed a serine (S) or a tyrosine (Y), and the ECSA and ECSA-IOL genotypes were characterized by a threonine (T). Interestingly, the Western African genotype displayed also an alanine (A) at this position. The position 225 was also discriminating the African genotypes characterized by an alanine (A) from the Asian genotypes, which displayed a serine (S). The position 226 is known for discriminating the ECSA and ECSA-IOL genotypes with an alanine (A) and a valine (V), respectively. The Asian genotypes, i.e. Asian and Asian-Pacific, displayed an alanine (A) at this position.

**Table 2:** Polymorphism analysis of the chikungunya E1 sequences.

Genotype	N	h	S	η	π	θ	ηs	ηp	Ka/Ks	Tajima’s D	Fu & Li’s D*	Fu & Li’s F*
ECSA	6	5	27	27	0.01719	0.01480	5	22	0.082	1.01925	1.11048	1.19180
ECSA-IOL	10	8	10	11	0.00292	0.00487	9	1	0.030	-1.79074*	-1.93522	-2.13705
Asian	7	6	25	25	0.01109	0.01277	16	9	0.070	-0.74824	-0.56450	-0.61115
Asian-Pacific	98	50	64	65	0.00845	0.01579	30	34	0.036	-1.50497	-3.25593*	-3.04089**

*Significant at 0.05.

**Significant at 0.02.

N, Number of samples; h, Number of haplotypes; S, Number of polymorphic sites; η, (Eta) Number of mutations; π, (Pi) Polymorphism at the nucleotide level; θ, (Theta) Watterson estimator of polymorphism (from Eta); ηs, Number of singletons; ηp, Number of parsimony significant sites.

## Discussion

CHIKV has been introduced in Asia at two different times. The first occurrence corresponded in the late 1950s to the deployment of the ECSA genotype (East/Central/South Africa), which gave rise to the Asian genotype (Hammon et al., 1960). The second occurrence was also due to the ECSA genotype, which in 2004 yielded the A226V mutant, also known as the Indian Ocean Lineage or IOL, which invaded the Indian Ocean in 2005 before spreading to India, Thailand, Cambodia, Singapore and Malaysia (Sam et al., 2009; Hapuarachchi et al., 2010; Rianthavorn et al., 2010; Tsetsarkin et al., 2011; Duong et al., 2012). However, the dynamic of CHIKV was not clearly established in Indonesia, which has been exposed to several chikungunya outbreaks over the past decades (MoH Indonesia, 2007; Harapan et al., 2019). This work brings some clarification on this dynamic.

The first conclusion is that a replacement of the Asian genotype of CHIKV by the Asian-Pacific genotype occurred in Indonesia between 1985 and 2000. This replacement is visible in both mosquitoes (our work) and humans (references and our work). Unfortunately, there is no sequence available from the 1985-2000 period to narrow down the time of occurrence of the replacement and try to identify the causes. No chikungunya cases were reported in Indonesia between 1985 and 2000. Until 1985, all CHIKVs sequenced in Indonesia belonged to the Asian genotype. This Asian genotype was present in several Asian countries. However, after 2000 almost exclusively the Asian-Pacific genotype was present in Indonesia. It is found all over the country until now. Few ECSA-IOL samples were detected but no Asian genotype could be found in databases after 2000. The Asian-Pacific genotype expanded in the Caribbean, South America, and Polynesia in 2013-2014, starting a pandemic (Lanciotti and Valadere, 2014; Leparc-Goffart et al., 2014; Nhan et al., 2014; Aubry et al., 2015; Diaz-Quinonez et al., 2016; Lanciotti and Lambert, 2016; Freitas et al., 2018; Wimalasiri-Yapa et al., 2019). The Caribbean isolates were found to be closer to the Yap Island isolates (Lanciotti and Valadere, 2014). The CHIKV contamination in French Polynesia was traced back to the Caribbean (Nhan et al., 2014) indicating a worldwide human-borne mobility. These massive Caribbean, South American and Polynesian epidemics were caused by what was initially described as an Asian genotype (Lanciotti and Valadere, 2014; Nhan et al., 2014). However, it is not the Asian genotype present since the 1960s that was responsible, but instead the distinct genotype we characterized as the Asian-Pacific genotype. This genotype was already present in Indonesia, and perhaps in other Asian-Pacific countries, long before the 2013 Caribbean and Polynesian outbreaks. It is not clear where this genotype appeared at the first place, in Yap Island or Micronesia, in the Pacific area or in Asia. Interestingly, this Asian-Pacific genotype developed in Indonesia while at the same time other Asian countries, mostly continental, i.e. India, Thailand, Cambodia, Singapore and Malaysia, were invaded by the ECSA-IOL genotype (Sam et al., 2009; Hapuarachchi et al., 2010; Rianthavorn et al., 2010; Tsetsarkin et al., 2011; Duong et al., 2012; Fu et al., 2019; Intayot et al., 2019; Zakotnik et al., 2019; Chansaenroj et al., 2020; Pyke et al., 2020). The reason for this duality is not known but would be worth investigating. A systematic review conducted in 2019 on CHIKV infection in Indonesia showed that out 130 sequences available, 120 (92.3%) were of what the authors named the Asian genotype and 10 (7.7%) belonged to the ECSA-IOL genotype (Harapan et al., 2019). All ECSA-IOL viruses in Indonesia were closely related to other CHIKV outbreaks in Southeast Asia countries during the same period but did not last beyond 2011 (Harapan et al., 2019). This is in agreement with the data reported in this work that indicate that the ECSA-IOL lineage was imported in Indonesia in 2009 through introduction for other countries but was never able to establish. It was quickly outcompeted by the Asian-Pacific genotype. However the reason for this lack of establishment of the ECSA-IOL genotype is not known. It would be important for the management of this disease to identify the drivers leading for the expansion of ECSA-IOL in one part of Asia and of the Asian-Pacific genotype in the other part.

The invasive capacity of the Asian-Pacific genotype demonstrated in Indonesia, but also in the Caribbean, South America and Polynesia (Lanciotti and Valadere, 2014; Leparc-Goffart et al., 2014; Nhan et al., 2014; Aubry et al., 2015; Diaz-Quinonez et al., 2016; Lanciotti and Lambert, 2016; Freitas et al., 2018; Wimalasiri-Yapa et al., 2019) raises the question of its selective advantage. The homogeneity of the Asian-Pacific genotype and its characterization as a population in expansion following a selective sweep along with its almost exclusive presence in Indonesia, its fast expansion in other continents and the absence of the Asian genotype in Indonesia strongly suggest the existence of a selective advantage. The presence of a selected marker at position 145 is certainly an indication of this pressure. However, it is not possible to determine at this stage what this selective advantage is. A higher transmissibility is a likely hypothesis but further analyses are needed to formally establish what this selective advantage could be. The invasiveness of IOL was due to a higher affinity for *Ae. albopictus* and a wide transportation by this mosquito species through international trade (Benedict et al., 2007). IOL showed invasiveness in the Indian Ocean and in Continental South and Southeast Asia where it replaced the initial Asian genotype (Sam et al., 2009; Hapuarachchi et al., 2010; Rianthavorn et al., 2010; Tsetsarkin et al., 2011; Duong et al., 2012; Quyen et al., 2017). Conversely, the invasiveness of the Asian-Pacific genotype does not seem to be related to the mosquito species since it can be transmitted by *Ae. aegypti, Ae. albopictus, Ae. hensilli, Ae. polynesiensis* and, perhaps by *Ae. butleri*, making it at higher risk of global dispersion. However, the presence of CHIKV in *Ae. butleri*, which is not a recognized vector, is not a proof of a transmission. Further analyses, such as testing saliva samples (Hall-Mendelin et al., 2010) would help determining the actual status of *Ae. butleri* as a vector of CHIKV.

The diversity of vectors was also found to be very low in Indonesia. *Aedes aegypti* samples, whether CHIKV-positive or not, belonged to the same population. This suggests that in parallel to the uniformity of CHIKV in Indonesia, there might also be a uniformity of *Ae. aegypti* vectors with a single population occupying the country. However, the number of samples is not high enough to bring a definitive conclusion and a larger genotyping study must be conducted at the scale of the whole country to determine the structure of the *Ae. aegypti* populations. This dynamic of *Aedes* vectors is not only of importance for chikungunya but also for dengue, Zika fever and other arbovirus diseases transmitted by the same mosquito species. Another issue raised by this work is the confirmation of the vertical transmission of CHIKV in *Ae. aegypti* under natural conditions. This had already been reported in *Ae. aegypti* populations from Delhi and Haryana states in India (Jain et al., 2016). This work is therefore a confirmation of the occurrence of natural vertical transmission in *Ae. aegypti* and it is the first report of natural vertical transmission of CHIKV in *Ae. albopictus*. Natural vertical transmission in *Ae. albopictus* was suspected but not demonstrated (Grandadam et al., 2011; Bellini et al., 2012). Previous works have investigated the rate of vertical transmission in *Ae. aegypti* and *Ae. albopictus* under experimental conditions (Agarwal et al., 2014; Chompoosri et al., 2016; Honório et al., 2019) but it is not possible to compare these data with those reported in this work under natural conditions. However, since all *Ae. aegypti* and *Ae. albopictus* mosquitoes analyzed in this work were adults having emerged from collected larvae, the calculated IRs, i.e. 0.24% and 0.23% for *Ae. aegypti* and *Ae. albopictus*, respectively, also correspond to the observed vertical transmission rate. The natural vertical transmission of CHIKV is an important aspect to consider in the dynamic of chikungunya fever.

The Asian-Pacific genotype of CHIKV described in Indonesia raises the question of the mechanisms behind this fast and massive replacement. Further studies must be conducted on the impact of the observed mutations on the rate of transmissibility. Societal aspects, which could favor the expansion of the virus, should also be investigated. Indonesia being an archipelago with no permanent contact between populations from different islands, trade and people mobility must be analyzed. Although IOL invaded largely Continental South and Southeast Asia owing to intensive international trade and people mobility, it does not seem to have invaded Indonesia. Further studies are therefore needed to understand why, unlike what happened on the continent, it is the Asian-Pacific genotype that expanded and is now dominating in Indonesia. Beyond chikungunya, these investigations will be useful to understand the dynamic of other arbovirus diseases, such as dengue, which are among the most widely distributed and fast-spreading diseases worldwide.

## Data Availability Statement

The datasets presented in this study can be found in online repositories. The names of the repository/repositories and accession number(s) can be found below: https://www.ncbi.nlm.nih.gov/genbank/, MW265951 to MW265970, https://www.ncbi.nlm.nih.gov/genbank/, MW270068 to MW270085, https://www.ncbi.nlm.nih.gov/genbank/, MW270131 to MW270147.

## Ethics Statement

The studies involving human participants were reviewed and approved by Health research ethics committee, national institute of health research and development (HREC-NIHRD), Ministry of Health of Republic of Indonesia. The patients/participants provided their written informed consent to participate in this study.

## Author Contributions

YA, TG, MH, MP, DP, SH, and TS conceived and designed the field studies. YA, TG, MH, MP, AY, KN, DP, and SH prepared samples. YA, MP, TG, and KN ran molecular analyses and laboratory experiments. TG, LG, and RF analyzed the data. TG and RF wrote the manuscript. SM and LG provided critics and significant revisions to the manuscript. All authors contributed to the article and approved the submitted version.

## Funding

The research was supported by the Institute for Vector and Reservoir Control Research and Development (IVRCRD), National Institute of Health Research and Development (NIHRD), Ministry of Health of the Republic of Indonesia (MoH RI). LG, SM, and RF were supported by the Université de Montpellier, IRD, ISEM, and CIRAD, Montpellier, France and by the French-Indonesian PHC Nusantara projects Zika &Co and SOCIAL.

## Conflict of Interest

The authors declare that the research was conducted in the absence of any commercial or financial relationships that could be constructed as a potential conflict of interest.

## Supplementary Material

Supplementary Figure 1 Map of the sampling locations in Indonesia.

Supplementary Figure 2 cox1 gene phylogeny of *Aedes aegypti* and *Aedes albopictus* mosquitoes. The tree was built using the maximum-likelihood (ML) method under the GTR+G model with 1,000 bootstrap repeats. Red: CHIKV-positive *Ae. albopictus* samples; Green: CHIKV-negative *Ae. albopictus* samples; Blue: CHIKV-positive *Ae. aegypti* samples; Purple: CHIKV-negative *Ae. aegypti* samples. The tree was rooted on the cox1 gene of *Culex quinquefasciatus* used as outgroup (beige).

Supplementary Figure 3 Phylogenetic analysis of the partial E1 gene of chikungunya viruses. The tree was built using the maximum-likelihood (ML) method under the GTR model with 1,000 bootstrap repeats. The tree was rooted on the E1 gene of the O’nyong-nyong virus (HM045785) used as outgroup. Purple: West African genotype; Dark blue: ECSA genotype (ECSA + ECSA-IOL); Orange: Asian genotype; Dark green: Asian-Pacific genotype samples from this work; Light green: Reference Asian-Pacific sequences.

Supplementary Figure 4 Phylogenetic analysis of the full-length E1 gene of chikungunya viruses. The tree was built using the maximum-likelihood (ML) method under the GTR model with 1,000 bootstrap repeats. The tree was rooted on the E1 gene of the O’nyong-nyong virus (HM045785) used as outgroup. Purple: West African genotype; Light blue: ECSA genotype; Dark blue: ECSA-IOL genotype; Orange: Asian genotype; Green: Asian-Pacific genotype.

Supplementary Figure 5 Haplotype network of the full-length E1 gene. The haplotype relationship was computed and constructed as a distance map based on median-joining network using PopART version 1.7. Purple: West African genotype; Light blue: ECSA genotype; Dark blue: ECSA-IOL genotype; Orange: Asian genotype; Green: Asian-Pacific genotype.

Supplementary Figure 6 Distribution tree of the full-length E1 protein sequence of chikungunya viruses. The tree was built using the maximum-likelihood (ML) method under the LG model with 500 bootstrap repeats. The tree was rooted on the E1 protein of the O’nyong-nyong virus (HM045785) used as outgroup. Purple: West African genotype; Light blue: ECSA genotype; Dark blue: ECSA-IOL genotype; Orange: Asian genotype; Green: Asian-Pacific genotype.

Supplementary Figure 7 Alignment of the full-length E1 protein sequences of chikungunya viruses.
