# Supplementary figures and images for: Fast Expansion of the Asian-Pacific Genotype of the Chikungunya Virus in Indonesia
Source: Front Cell Infect Microbiol. 2021 Apr 21;11:631508. doi: 10.3389/fcimb.2021.631508 (PMC8098665; doi:10.3389/fcimb.2021.631508)

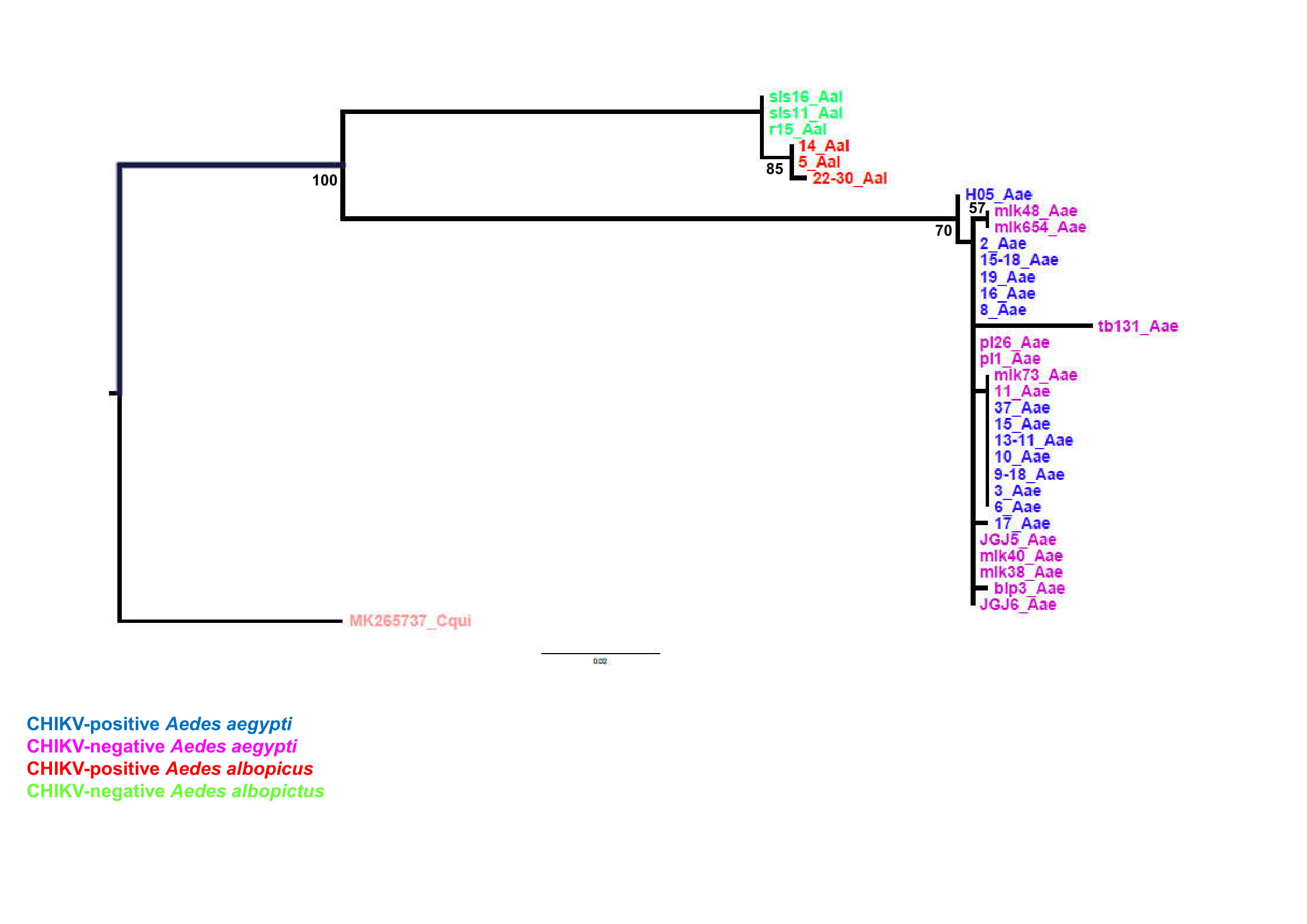

Supplement: Supplementary Figure 2 — cox1 gene phylogeny of Aedes aegypti and Aedes albopictus mosquitoes. The tree was built using the maximum-likelihood (ML) method under the GTR+G model with 1,000 bootstrap repeats. Red: CHIKV-positive Ae. albopictus samples; Green: CHIKV-negative Ae. albopictus samples; Blue: CHIKV-positive Ae. aegypti samples; Purple: CHIKV-negative Ae. aegypti samples. The tree was rooted on the cox1 gene of Culex quinquefasciatus used as outgroup (beige). [file Image_2.jpeg]

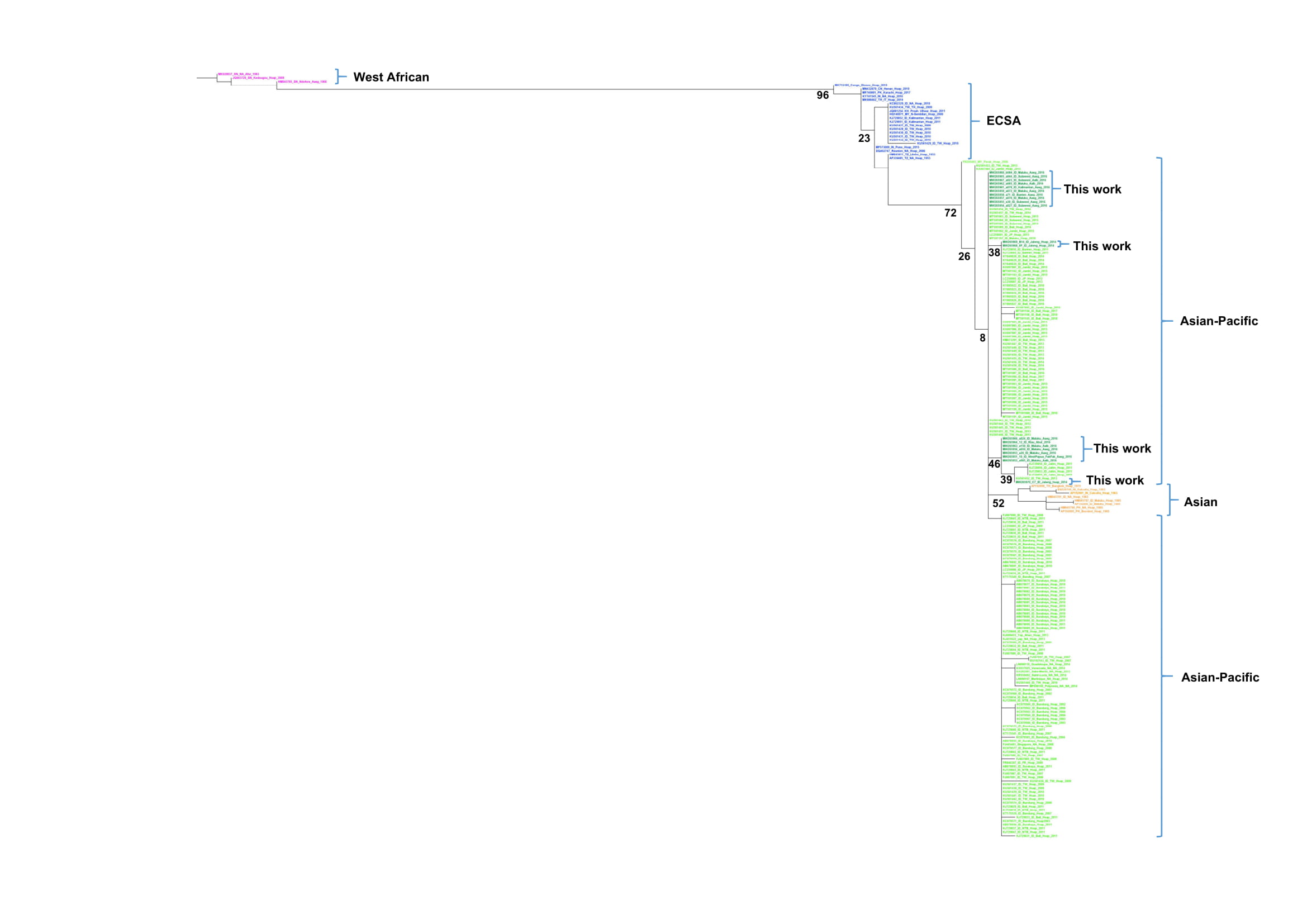

Supplement: Supplementary Figure 3 — Phylogenetic analysis of the partial E1 gene of chikungunya viruses. The tree was built using the maximum-likelihood (ML) method under the GTR model with 1,000 bootstrap repeats. The tree was rooted on the E1 gene of the O’nyong-nyong virus (HM045785) used as outgroup. Purple: West African genotype; Dark blue: ECSA genotype (ECSA + ECSA-IOL); Orange: Asian genotype; Dark green: Asian-Pacific genotype samples from this work; Light green: Reference Asian-Pacific sequences. [file Image_3.jpeg]

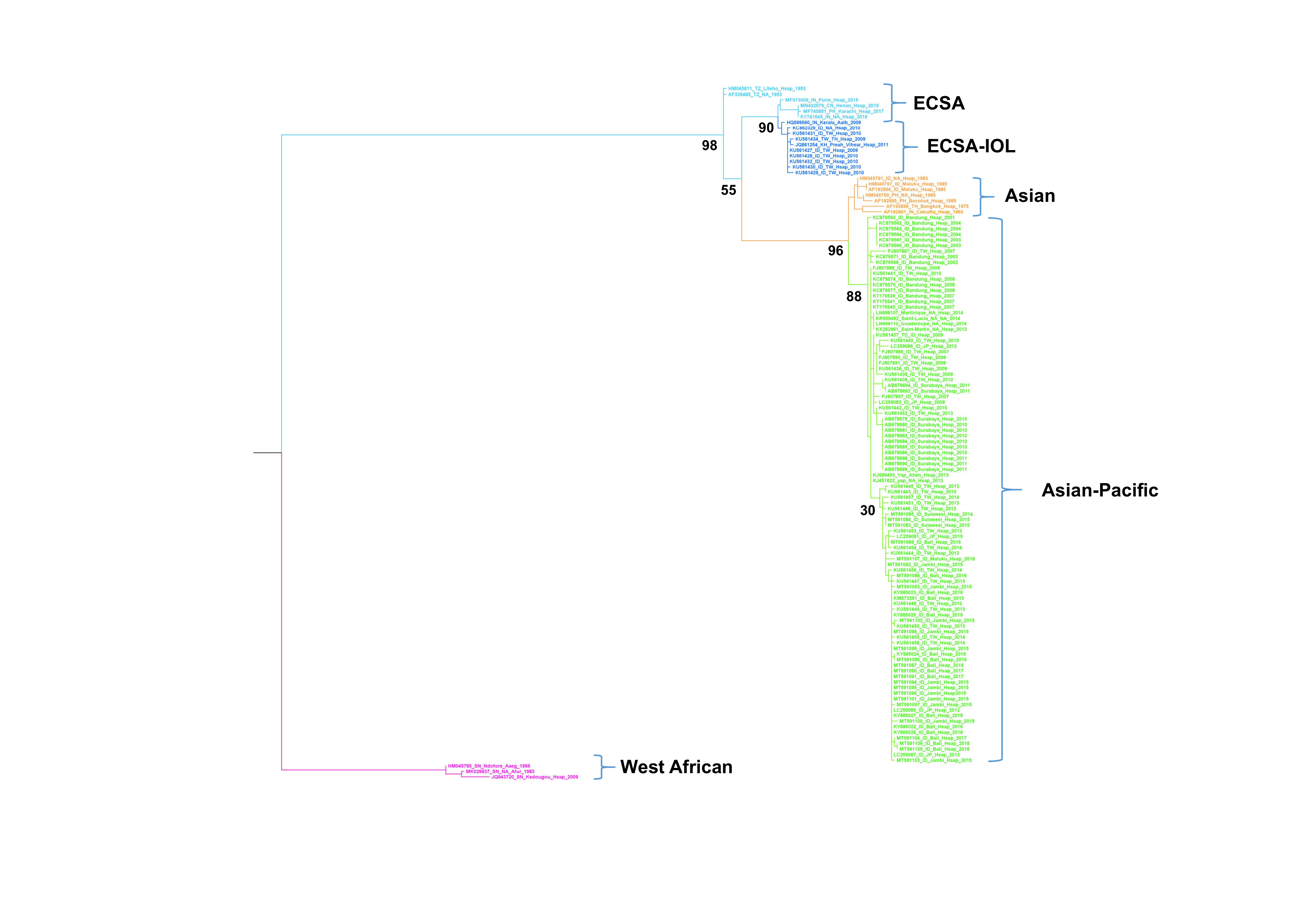

Supplement: Supplementary Figure 4 — Phylogenetic analysis of the full-length E1 gene of chikungunya viruses. The tree was built using the maximum-likelihood (ML) method under the GTR model with 1,000 bootstrap repeats. The tree was rooted on the E1 gene of the O’nyong-nyong virus (HM045785) used as outgroup. Purple: West African genotype; Light blue: ECSA genotype; Dark blue: ECSA-IOL genotype; Orange: Asian genotype; Green: Asian-Pacific genotype. [file Image_4.jpeg]

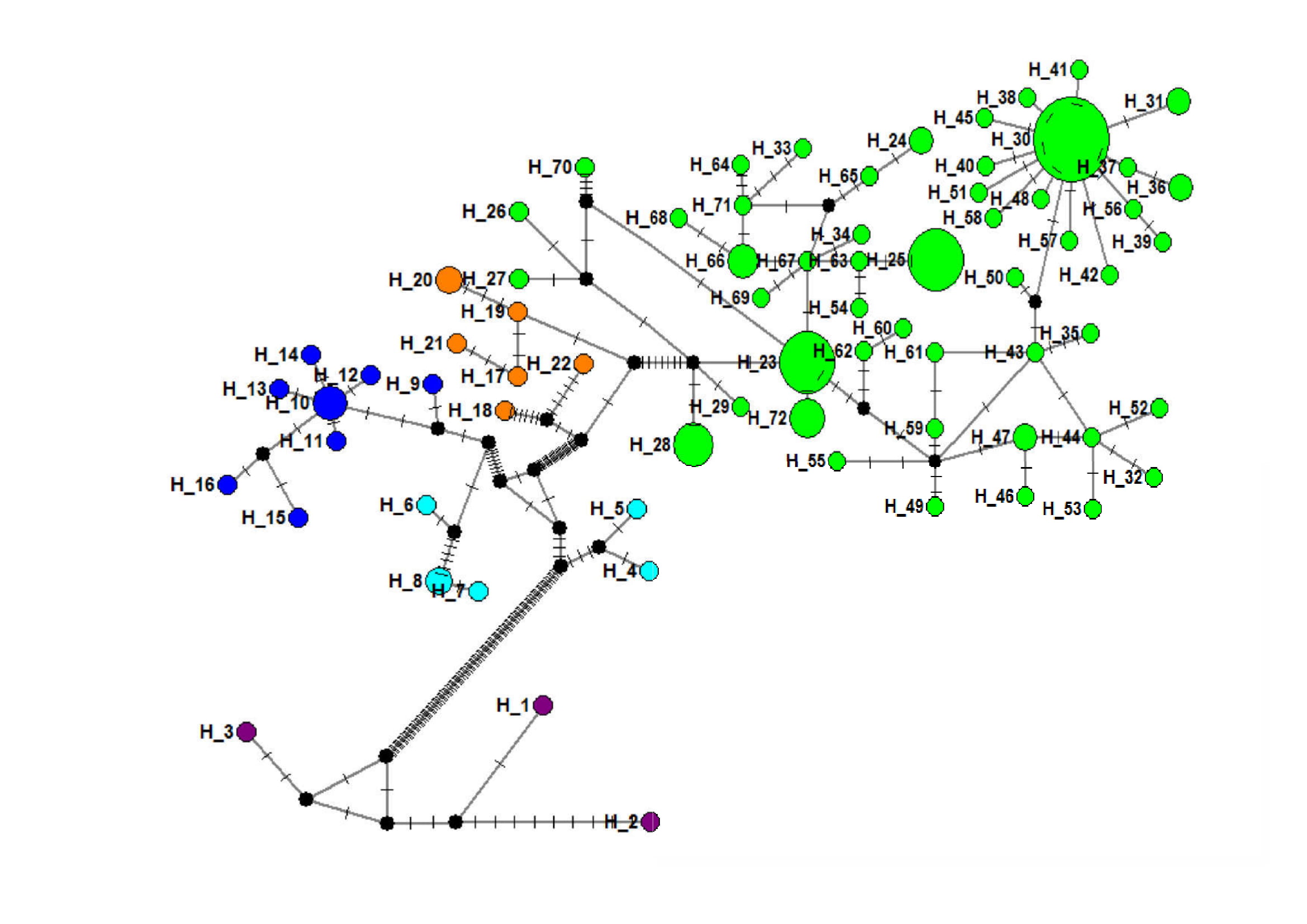

Supplement: Supplementary Figure 5 — Haplotype network of the full-length E1 gene. The haplotype relationship was computed and constructed as a distance map based on median-joining network using PopART version 1.7. Purple: West African genotype; Light blue: ECSA genotype; Dark blue: ECSA-IOL genotype; Orange: Asian genotype; Green: Asian-Pacific genotype. [file Image_5.jpeg]

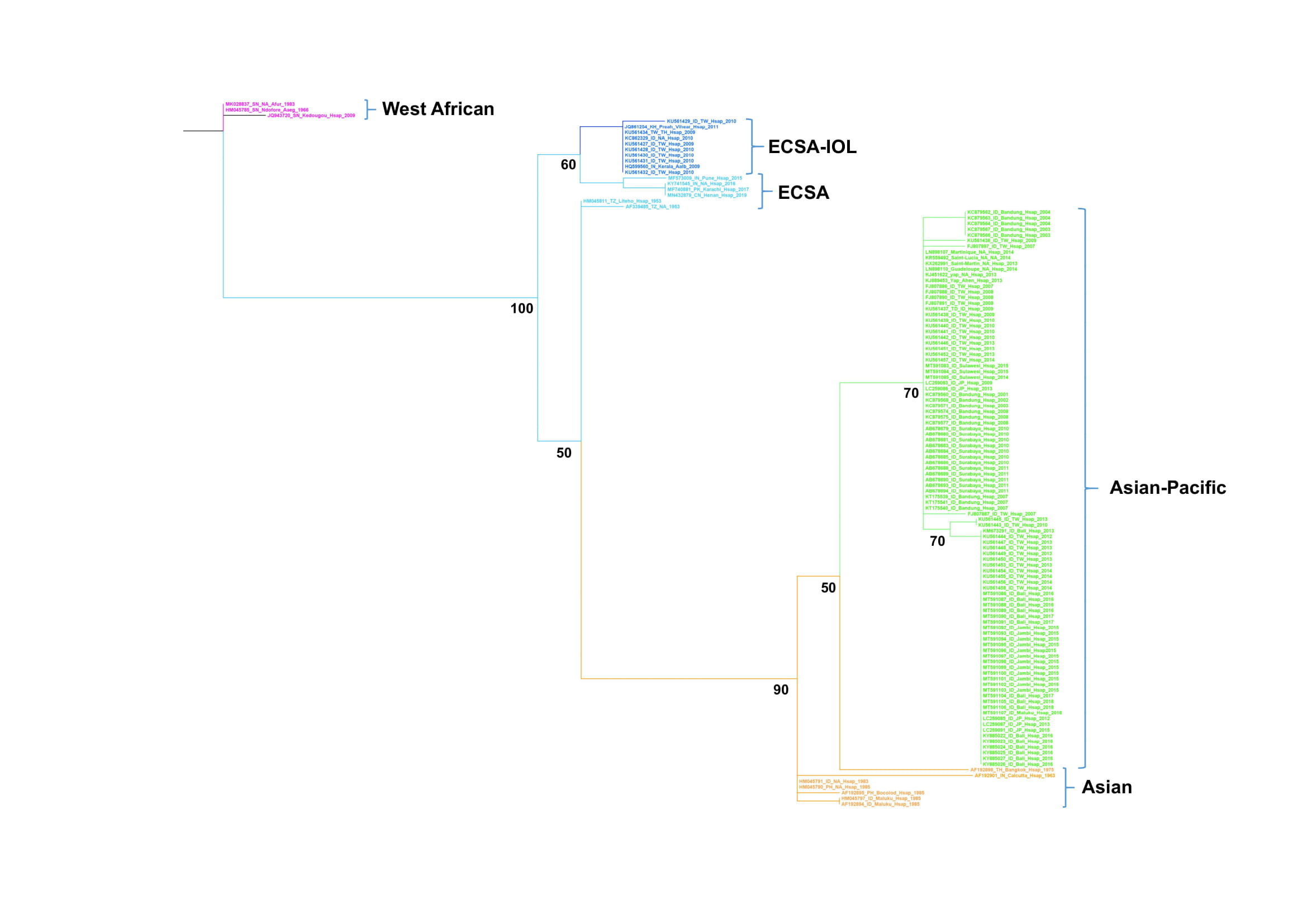

Supplement: Supplementary Figure 6 — Distribution tree of the full-length E1 protein sequence of chikungunya viruses. The tree was built using the maximum-likelihood (ML) method under the LG model with 500 bootstrap repeats. The tree was rooted on the E1 protein of the O’nyong-nyong virus (HM045785) used as outgroup. Purple: West African genotype; Light blue: ECSA genotype; Dark blue: ECSA-IOL genotype; Orange: Asian genotype; Green: Asian-Pacific genotype. [file Image_6.jpeg]

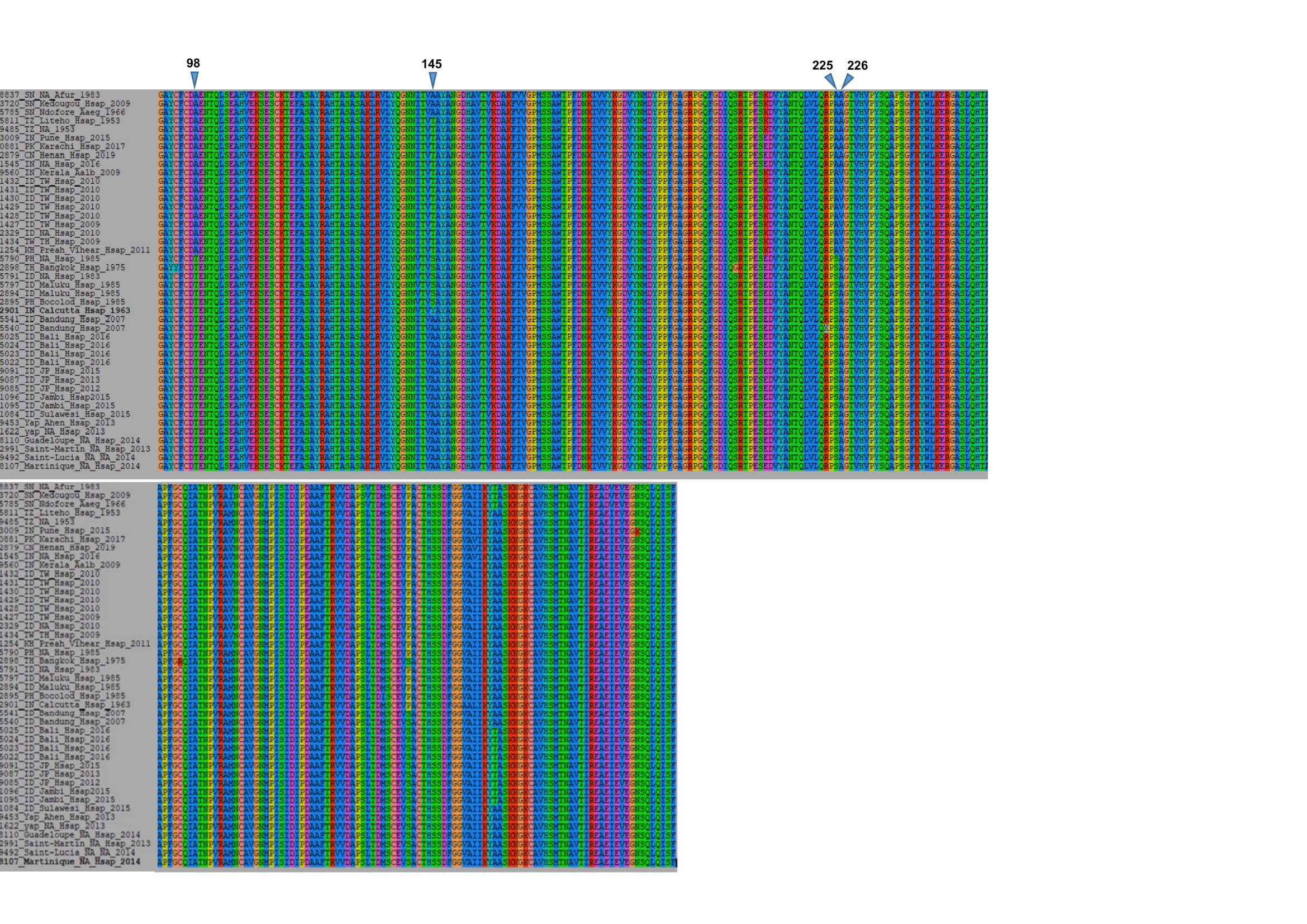

Supplement: Supplementary Figure 7 — Alignment of the full-length E1 protein sequences of chikungunya viruses. [file Image_7.jpeg]
