# Supplementary material for: Fast Expansion of the Asian-Pacific Genotype of the Chikungunya Virus in Indonesia
Source: Front Cell Infect Microbiol. 2021 Apr 21;11:631508. doi: 10.3389/fcimb.2021.631508 (PMC8098665; doi:10.3389/fcimb.2021.631508)
Supplement: Supplementary file 8 [file Table_1.docx]

**Supplementary Table 1. Mosquito collection and chikungunya virus positivity**

**Province Location Species Number Number of Number of Number of**

**of pools mosquitoes positive pools positive mosquitoes**

Banten Pandeglang *Ae. aegypti* 11 33 0 0

*Ae. albopictus* 2 7 0 0

Banten Lebak *Ae. aegypti* 9 22 1 1

*Ae. albopictus* 51 353 0 0

Banten Serang *Ae. aegypti* 4 7 0 0

*Ae. albopictus* 11 108 0 0

Riau Bengkalis *Ae. aegypti* 3 5 0 0

*Ae. albopictus* 55 261 0 0

*Ae. butleri* 36 165 1 1

Riau Meranti *Ae. aegypti* 24 132 0 0

*Ae. albopictus* 14 141 0 0

Riau Dumai *Ae. aegypti* 23 115 0 0

*Ae. albopictus* 13 82 0 0

Yogyakarta Kulon Progo *Ae. aegypti* 0 0 0 0

*Ae. albopictus* 12 68 0 0

Yogyakarta Bantul *Ae. aegypti* 29 124 1 1

*Ae. albopictus* 9 79 0 0

Yogyakarta Gunung Kidul *Ae. aegypti* 2 2 0 0

*Ae. albopictus* 31 163 0 0

Central Kalimantan Gunung Mas Ae. aegypti 24 146 0 0

*Ae. albopictus* 21 66 0 0

Central Kalimantan Murung Raya *Ae. aegypti* 2 8 0 0

*Ae. albopictus* 7 54 0 0

Central Kalimantan Pulang Pisau *Ae. aegypti* 33 122 1 4

*Ae. albopictus* 6 23 0 0

Southeast Sulawesi Muna *Ae. aegypti* 148 1042 5 5

*Ae. albopictus* 10 56 0 0

Southeast Sulawesi Konawe *Ae. aegypti* 18 108 0 0

*Ae. albopictus* 1 25 0 0

Southeast Sulawesi Bombana *Ae. aegypti* 33 632 0 0

*Ae. albopictus* 0 0 0 0

Maluku West Southwest *Ae. aegypti* 19 274 0 0

Maluku *Ae. albopictus* 2 4 1 1

Maluku Southeast Maluku *Ae. aegypti* 200 4709 6 6

*Ae. albopictus* 38 913 2 2

Maluku Aru Islands *Ae. aegypti* 96 1732 0 0

*Ae. albopictus* 4 55 0 0

North Maluku Central Halmahera *Ae. aegypti* 5 117 0 0

*Ae. albopictus* 3 44 0 0

North Maluku South Halmahera *Ae. aegypti* 73 1800 10 10

*Ae. albopictus* 32 716 1 1

North Maluku Morotai Islands *Ae. aegypti* 5 16 0 0

*Ae. albopictus* 22 157 4 4

West Papua Manokwari *Ae. aegypti* 9 142 0 0

*Ae. albopictus* 5 23 0 0

West Papua Fak-Fak *Ae. aegypti* 30 488 1 1

*Ae. albopictus*  5 23 0 0

Total *Ae. aegypti* 800 11776 25 28

Total *Ae. albopictus* 354 3421 8 8

**Total^a^ 1154 15197 33 36**
