# Supplementary material for: Fast Expansion of the Asian-Pacific Genotype of the Chikungunya Virus in Indonesia
Source: Front Cell Infect Microbiol. 2021 Apr 21;11:631508. doi: 10.3389/fcimb.2021.631508 (PMC8098665; doi:10.3389/fcimb.2021.631508)
Supplement: Supplementary file 9 [file Table_2.docx]

**Supplementary Table 2. Chikungunya virus sequences used for phylogenetic analysis**

**Isolate Location Year Host Genotype Accession #**

PM2951 Senegal 1966 *Ae. aegypti* West African HM045785

Senegal/1983/37997 Senegal 1983 *Ae. furcifer* West African MK028837

B013909 Senegal 2009 Human West African JQ943720

Ross low-psg Tanzania 1953 Human ECSA HM045811

S27 Tanzania 1953 Human ECSA AF339485

IND-2015-MH_Pune1517011 India 2015 Human ECSA MF573009

Pakistan-10 Pakistan 2017 Human ECSA MF740881

CHIKV/Henan001/2019 China 2019 Human ECSA MN432879

ILBS2 India 2016 Human ECSA KY741545

PF2219-INMI Italy/Thailand 2019 Human ECSA MK986662

CHK-Kalbar.INA:2011*075 Indonesia 2011 Human ECSA* KJ729851

CHK-Kalbar.INA:2011*077 Indonesia 2011 Human ECSA* KJ729852

216-NSembilan-2009 Malaysia 2009 Human ECSA* HQ148971

RC_Diosso_2019_31 Congo 2019 Human ECSA-IOL MK715490

IMT/6470 Reunion Island 2006 Human ECSA-IOL DQ462747

0911aTw Thailand 2009 Human ECSA-IOL KU561434

V1024308_KH11_PVH Cambodia 2011 Human ECSA-IOL JQ861254

1006aTw Indonesia/Taiwan 2010 Human ECSA-IOL KU561432

1003aTw Indonesia/Taiwan 2010 Human ECSA-IOL KU561431

1002dTw Indonesia/Taiwan 2010 Human ECSA-IOL KU561430

1002cTw Indonesia/Taiwan 2010 Human ECSA-IOL KU561429

1002aTw Indonesia/Taiwan 2010 Human ECSA-IOL KU561428

0912aTw Indonesia/Taiwan 2009 Human ECSA-IOL KU561427

NL10/152 NA, Indonesia 2010 Human ECSA-IOL KC862329

KC28 India *Ae. albopictus* ECSA-IOL HQ599560

JKT23574 Indonesia 1983 Human Asian HM045791

RSU1 Indonesia 1985 Human Asian HM045797

RSU1 Indonesia 1985 Human Asian AF192894

PH H15483 Philippines 1985 Human Asian AF192895

1455/75 Thailand 1975 Human Asian AF192898

Gibbs 63-263 India 1963 Human Asian AF192901

IND63WB1 India 1963 Human Asian DQ520746

Ph15483 Philippines 1985 Human Asian HM045790

CHIKV/Homo sapiens/SXM/

H-20235-STMARTIN-2013/2003 Saint Martin 2013 Human Asian-Pacific KX262991

WHCHK23 Saint Lucia 2014 Human Asian-Pacific KR559492

LARD810 Venezuela 2014 Human Asian-Pacific KX037025

M108 Martinique 2014 Human Asian-Pacific LN898107

G106 Guadeloupe 2014 Human Asian-Pacific LN898110

NA French Polynesia 2014 NA Asian-Pacific MF696160

3807 Yap, Micronesia 2013 Human Asian-Pacific KJ451622

Yap 13-2148 Yap, Micronesia 2013 *Ae. hensilli* Asian-Pacific KJ689453

SGEHICHS412308 Indonesia/Singapore 2008 Human Asian-Pacific FJ445483

Pt11352 Indonesia/France 2009 Human Asian-Pacific FR846307

0804aTw Indonesia/Taiwan 2008 Human Asian-Pacific FJ807889

0706aTw Indonesia/Taiwan 2007 Human Asian-Pacific EU192143

MY/06/37348 Malaysia 2006 Human Asian-Pacific FN295483

CHK-Banten.INA:2011*066 Banten, Indonesia 2011 Human Asian-Pacific KJ729849

CHK-Banten.INA:2011*068 Banten, Indonesia 2011 Human Asian-Pacific KJ729850

CHK-Bali.INA:2011*016 Bali, Indonesia 2011 Human Asian-Pacific KJ729836

CHK-Bali.INA:2011*014 Bali, Indonesia 2011 Human Asian-Pacific KJ729835

2001918633-BDG Bandung, Indonesia 2001 Human Asian-Pacific KC879561

2001908323-BDG Bandung, Indonesia 2001 Human Asian-Pacific KC879559

2007904923-BDG Bandung, Indonesia 2007 Human Asian-Pacific KC879578

2003902452-BDG Bandung, Indonesia 2003 Human Asian-Pacific KC879570

2008900345-BDG Bandung, Indonesia 2008 Human Asian-Pacific KC879573

2008900207-BDG Bandung, Indonesia 2008 Human Asian-Pacific KC879576

2002918314-BDG Bandung, Indonesia 2002 Human Asian-Pacific KC879569

2004904879-BDG Bandung, Indonesia 2003 Human Asian-Pacific KC879565

CHK-Jatim.INA:2011*108 East Java, Indonesia 2011 Human Asian-Pacific KJ729856

CHK-Jatim.INA:2011*107 East Java, Indonesia 2011 Human Asian-Pacific KJ729855

CHK-Jatim.INA:2011*105 East Java, Indonesia 2011 Human Asian-Pacific KJ729854

CHK-Jatim.INA:2011*096 East Java, Indonesia 2011 Human Asian-Pacific KJ729853

CHIK/SBY10/10 Surabaya, Indonesia 2010 Human Asian-Pacific AB678678

CHIK/SBY8/10 Surabaya, Indonesia 2010 Human Asian-Pacific AB678677

CHIK/SBY6/10 Surabaya, Indonesia 2010 Human Asian-Pacific AB678691

CHK-NTB.INA:2011*064 West Nusa Tenggara, Indonesia 2011 Human Asian-Pacific KJ729848

CHK-NTB.INA:2011*048 West Nusa Tenggara, Indonesia 2011 Human Asian-Pacific KJ729841

JMB-230 Jambi, Indonesia 2015 Human Asian-Pacific KX097988.

JMB-192 Jambi, Indonesia 2015 Human Asian-Pacific KX097986

JMB-154 Jambi, Indonesia 2015 Human Asian-Pacific KX097982

JMB-205 Jambi, Indonesia 2015 Human Asian-Pacific KX097987

JMB-187 Jambi, Indonesia 2015 Human Asian-Pacific KX097985

JMB-172 Jambi, Indonesia 2015 Human Asian-Pacific KX097984

JMB-167 Jambi, Indonesia 2015 Human Asian-Pacific KX097983

JMB-015 Jambi, Indonesia 2015 Human Asian-Pacific KX097981

GianyarBali-4-E1 Bali, Indonesia 2014 Human Asian-Pacific KY649630

GianyarBali-3-E1 Bali, Indonesia 2014 Human Asian-Pacific KY649629

GianyarBali-1-E1 Bali, Indonesia 2014 Human Asian-Pacific KY649628

A023 Muna, Indonesia 2016 *Ae. albopictus* Asian-Pacific MW265967 (This work)

a027 Muna, Indonesia 2016 *Ae. aegypti* Asian-Pacific MW265954 (This work)

a30 Muna, Indonesia 2016 *Ae. aegypti* Asian-Pacific MW265955 (This work)

a044 Muna, Indonesia 2016 *Ae. aegypti* Asian-Pacific MW265965 (This work)

10 Fak Fak, Indonesia 2016 *Ae. aegypti* Asian-Pacific MW265951 (This work)

12 Bengkalis, Indonesia 2016 *Ae. butleri* Asian-Pacific MW265964 (This work)

85 Southeast Maluku, Indonesia 2016 *Ae. aegypti* Asian-Pacific MW265962 (This work)

A070 Southeast Maluku, Indonesia 2016 *Ae. aegypti* Asian-Pacific MW265957 (This work)

a150 Southeast Maluku, Indonesia 2016 *Ae. albopictus* Asian-Pacific MW265963 (This work)

b084 Southeast Maluku, Indonesia 2016 *Ae. aegypti* Asian-Pacific MW265960 (This work)

a20 Southeast Maluku, Indonesia 2016 *Ae. aegypti* Asian-Pacific MW265953 (This work)

a024 South Halmahera, Indonesia 2016 *Ae. aegypti* Asian-Pacific MW265966 (This work)

a050 South Halmahera, Indonesia 2016 *Ae. aegypti* Asian-Pacific MW265956 (This work)

a072 South Halmahera, Indonesia 2016 *Ae. aegypti* Asian-Pacific MW265959 (This work)

a085 South Halmahera, Indonesia 2016 *Ae. albopictus* Asian-Pacific MW265962 (This work)

a71 Lebak, Indonesia 2016 *Ae. aegypti* Asian-Pacific MW265958 (This work)

a078 Pulang Pisau, Indonesia 2016 *Ae. aegypti* Asian-Pacific MW265961 (This work)

8F Jateng, Indonesia 2014 Human Asian-Pacific MW265968 (This work)

B14 Jateng, Indonesia 2014 Human Asian-Pacific MW265969 (This work)

C7 Jateng, Indonesia 2014 Human Asian-Pacific MW265970 (This work)

CSB04010 Bandung, Indonesia 2007 Human Asian-Pacific KT175541

CSB04003 Bandung, Indonesia 2007 Human Asian-Pacific KT175540

CSB04001 Bandung, Indonesia 2007 Human Asian-Pacific KT175539

CHK-NTB.INA:2011*061 NTB, Indonesia 2011 Human Asian-Pacific KJ729847

CHK-NTB.INA:2011*059 NTB, Indonesia 2011 Human Asian-Pacific KJ729846

CHK-NTB.INA:2011*057 NTB, Indonesia 2011 Human Asian-Pacific KJ729845

CHK-NTB.INA:2011*056 NTB, Indonesia 2011 Human Asian-Pacific KJ729844

CHK-NTB.INA:2011*055 NTB, Indonesia 2011 Human Asian-Pacific KJ729843

CHK-NTB.INA:2011*052 NTB, Indonesia 2011 Human Asian-Pacific KJ729842

CHK-NTB.INA:2011*048 NTB, Indonesia 2011 Human Asian-Pacific KJ729841

CHK-NTB.INA:2011*047 NTB, Indonesia 2011 Human Asian-Pacific KJ729840

CHK-NTB.INA:2011*046 NTB, Indonesia 2011 Human Asian-Pacific KJ729839

CHK-NTB.INA:2011*044 NTB, Indonesia 2011 Human Asian-Pacific KJ729838

CHK-NTB.INA:2011*041 NTB, Indonesia 2011 Human Asian-Pacific KJ729837

CHK-Bali.INA:2011*016 NTB, Indonesia 2011 Human Asian-Pacific KJ729836

CHK-Bali.INA:2011*014 Bali, Indonesia 2011 Human Asian-Pacific KJ729835

CHK-Bali.INA:2011*013 Bali, Indonesia 2011 Human Asian-Pacific KJ729834

CHK-Bali.INA:2011*009 Bali, Indonesia 2011 Human Asian-Pacific KJ729833

CHK-Bali.INA:2011*008 Bali, Indonesia 2011 Human Asian-Pacific KJ729832

CHK-Bali.INA:2011*006 Bali, Indonesia 2011 Human Asian-Pacific KJ729831

CHK-Bali.INA:2011*004 Bali, Indonesia 2011 Human Asian-Pacific KJ729830

CHK-Bali.INA:2011*003 Bali, Indonesia 2011 Human Asian-Pacific KJ729829

CHIK/SBY79/10 Surabaya, Indonesia 2010 Human Asian-Pacific AB678694

CHIK/SBY59/10 Surabaya, Indonesia 2010 Human Asian-Pacific AB678693

CHIK/SBYK1Mos/11 Surabaya, Indonesia 2011 Human Asian-Pacific AB678690

CHIK/SBYC1Mos/11 Surabaya, Indonesia 2011 Human Asian-Pacific AB678689

CHIK/SBY83/11 Surabaya, Indonesia 2011 Human Asian-Pacific AB678688

CHIK/SBY183/10 Surabaya, Indonesia 2010 Human Asian-Pacific AB678686

CHIK/SBY95/10 Surabaya, Indonesia 2010 Human Asian-Pacific AB678685

CHIK/SBY86/10 Surabaya, Indonesia 2010 Human Asian-Pacific AB678684

CHIK/SBY76/10 Surabaya, Indonesia 2010 Human Asian-Pacific AB678683

CHIK/SBY53/10 Surabaya, Indonesia 2010 Human Asian-Pacific AB678681

CHIK/SBY13/10 Surabaya, Indonesia 2010 Human Asian-Pacific AB678680

CHIK/SBY11/10 Surabaya, Indonesia 2010 Human Asian-Pacific AB678679

2008900245-BDG Bandung, Indonesia 2008 Human Asian-Pacific KC879577

2008910293-BDG Bandung, Indonesia 2008 Human Asian-Pacific KC879575

2008910307-BDG Bandung, Indonesia 2008 Human Asian-Pacific KC879574

2003902453-BDG Bandung, Indonesia 2003 Human Asian-Pacific KC879571

2002918310-BDG Bandung, Indonesia 2002 Human Asian-Pacific KC879568

2003910438-BDG Bandung, Indonesia 2003 Human Asian-Pacific KC879567

2003909662-BDG Bandung, Indonesia 2003 Human Asian-Pacific KC879566

2004906079-BDG Bandung, Indonesia 2004 Human Asian-Pacific KC879564

2004906033-BDG Bandung, Indonesia 2004 Human Asian-Pacific KC879563

2004904899-BDG Bandung, Indonesia 2004 Human Asian-Pacific KC879562

2001907981-BDG Bandung, Indonesia 2001 Human Asian-Pacific KC879560

201610136 Bali, Indonesia 2016 Human Asian-Pacific KY885027

201610133 Bali, Indonesia 2016 Human Asian-Pacific KY885026

201610129 Bali, Indonesia 2016 Human Asian-Pacific KY885025

201610127 Bali, Indonesia 2016 Human Asian-Pacific KY885024

201610126 Bali, Indonesia 2016 Human Asian-Pacific KY885023

201610125 Bali, Indonesia 2016 Human Asian-Pacific KY885022

CHIKV/Hu/Indonesia/NIID35/2015 NA, Indonesia 2015 Human Asian-Pacific LC259091

CHIKV/Hu/Indonesia/NIID112/2013 NA, Indonesia 2013 Human Asian-Pacific LC259087

[CHIKV/Hu/Indonesia/NIID108/2013](https://www.ncbi.nlm.nih.gov/nuccore/LC259086.1) NA, Indonesia 2013 Human Asian-Pacific LC259086

CHIKV/Hu/Indonesia/NIID181/2012 NA, Indonesia 2012 Human Asian-Pacific LC259085

CHIKV/Hu/Indonesia/NIID58/2009 NA, Indonesia 2009 Human Asian-Pacific LC259083

AMB-041 Maluku, Indonesia 2018 Human Asian-Pacific MT591107

TBN-657 Bali, Indonesia 2018 Human Asian-Pacific MT591106

TBN-623 Bali, Indonesia 2018 Human Asian-Pacific MT591105

TBN-003 Bali, Indonesia 2017 Human Asian-Pacific MT591104

JMB-167 Bali, Indonesia 2015 Human Asian-Pacific MT591103

JMB-288 Jambi, Indonesia 2015 Human Asian-Pacific MT591102

JMB-164 Jambi, Indonesia 2015 Human Asian-Pacific MT591101

JMB-337 Jambi, Indonesia 2015 Human Asian-Pacific MT591100

JMB-351 Jambi, Indonesia 2015 Human Asian-Pacific MT591099

JMB-187 Jambi, Indonesia 2015 Human Asian-Pacific MT591098

JMB-334 Jambi, Indonesia 2015 Human Asian-Pacific MT591097

JMB-474 Jambi, Indonesia 2015 Human Asian-Pacific MT591096

JMB-209 Jambi, Indonesia 2015 Human Asian-Pacific MT591095

JMB-308 Jambi, Indonesia 2015 Human Asian-Pacific MT591094

JMB-331 Jambi, Indonesia 2015 Human Asian-Pacific MT591093

JMB-172 Jambi, Indonesia 2015 Human Asian-Pacific MT591092

TBN-103 Bali, Indonesia 2017 Human Asian-Pacific MT591091

TBN-017 Bali, Indonesia 2017 Human Asian-Pacific MT591090

201610125 Bali, Indonesia 2016 Human Asian-Pacific MT591089

201610136 Bali, Indonesia 2016 Human Asian-Pacific MT591088

201610133 Bali, Indonesia 2016 Human Asian-Pacific MT591087

201610127 Bali, Indonesia 2016 Human Asian-Pacific MT591086

TMH-073 Sulawesi, Indonesia 2014 Human Asian-Pacific MT591085

TMH-104 Sulawesi, Indonesia 2015 Human Asian-Pacific MT591084

TMH-092 Sulawesi, Indonesia 2015 Human Asian-Pacific MT591083

1408aTw Indonesia/Taiwan 2014 Human Asian-Pacific KU561458

1406aTw Indonesia/Taiwan 2014 Human Asian-Pacific KU561457

1404aTw Indonesia/Taiwan 2014 Human Asian-Pacific KU561456

1403bTw Indonesia/Taiwan 2014 Human Asian-Pacific KU561455

1403aTw Indonesia/Taiwan 2014 Human Asian-Pacific KU561454

1312cTw Indonesia/Taiwan 2013 Human Asian-Pacific KU561453

1310aTw Indonesia/Taiwan 2013 Human Asian-Pacific KU561452

1308cTw Indonesia/Taiwan 2013 Human Asian-Pacific KU561451

1308bTw Indonesia/Taiwan 2013 Human Asian-Pacific KU561450

1307aTw Indonesia/Taiwan 2013 Human Asian-Pacific KU561449

1304aTw Indonesia/Taiwan 2013 Human Asian-Pacific KU561448

1303aTw Indonesia/Taiwan 2013 Human Asian-Pacific KU561447

1302aTw Indonesia/Taiwan 2013 Human Asian-Pacific KU561446

1301aTw Indonesia/Taiwan 2013 Human Asian-Pacific KU561445

1207aTw Indonesia/Taiwan 2012 Human Asian-Pacific KU561444

1011aTw Indonesia/Taiwan 2010 Human Asian-Pacific KU561443

1006bTw Indonesia/Taiwan 2010 Human Asian-Pacific KU561442

1005bTw Indonesia/Taiwan 2010 Human Asian-Pacific KU561441

1005aTw Indonesia/Taiwan 2010 Human Asian-Pacific KU561440

1002bTw Indonesia/Taiwan 2010 Human Asian-Pacific KU561439

0909aTw Indonesia/Taiwan 2009 Human Asian-Pacific KU561438

0908aTw Indonesia/Taiwan 2009 Human Asian-Pacific KU561437

0904bTw Indonesia/Taiwan 2009 Human Asian-Pacific KU561436

DH130003 Bali, Indonesia 2013 Human Asian-Pacific KM673291

0706aTw Indonesia/Taiwan 2007 Human Asian-Pacific FJ807897

0811aTw Indonesia/Taiwan 2008 Human Asian-Pacific FJ807891

0806aTw Indonesia/Taiwan 2008 Human Asian-Pacific FJ807890

0802aTw Indonesia/Taiwan 2008 Human Asian-Pacific FJ807888

0712bTw Indonesia/Taiwan 2007 Human Asian-Pacific FJ807887

0712aTw Indonesia/Taiwan 2007 Human Asian-Pacific FJ807886

* ECSA sequences too short to identify the amino acid at position 226
